# Patient–Specific Immersed Finite Element–Difference Model of Transcatheter Aortic Valve Replacement

**DOI:** 10.1007/s10439-022-03047-3

**Published:** 2022-10-20

**Authors:** Jordan A. Brown, Jae H. Lee, Margaret Anne Smith, David R. Wells, Aaron Barrett, Charles Puelz, John P. Vavalle, Boyce E. Griffith

**Affiliations:** 1 Department of Mathematics, University of North Carolina, Chapel Hill, NC, USA; 2 University of North Carolina School of Medicine, Chapel Hill, NC, USA; 3 Division of Cardiology, Department of Pediatrics, Baylor College of Medicine and Texas Children’s Hospital, Houston, TX, USA; 4 Division of Cardiology, Department of Medicine, University of North Carolina School of Medicine, Chapel Hill, NC, USA; 5 Departments of Mathematics, Applied Physical Sciences, and Biomedical Engineering, University of North Carolina, Chapel Hill, NC, USA; 6 Carolina Center for Interdisciplinary Applied Mathematics, University of North Carolina, Chapel Hill, NC, USA; 7 Computational Medicine Program, University of North Carolina School of Medicine, Chapel Hill, NC, USA; 8 McAllister Heart Institute, University of North Carolina School of Medicine, Chapel Hill, NC, USA

**Keywords:** Transcatheter aortic valve replacement, Bioprosthetic heart valve, Immersed finite element-difference method, Finite element method

## Abstract

Transcatheter aortic valve replacement (TAVR) first received FDA approval for high-risk surgical patients in 2011 and has been approved for low-risk surgical patients since 2019. It is now the most common type of aortic valve replacement, and its use continues to accelerate. Computer modeling and simulation (CM&S) is a tool to aid in TAVR device design, regulatory approval, and indication in patient-specific care. This study introduces a computational fluid-structure interaction (FSI) model of TAVR with Medtronic’s *CoreValve Evolut R* device using the immersed finite element-difference (IFED) method. We perform dynamic simulations of crimping and deployment of the Evolut R, as well as device behavior across the cardiac cycle in a patient-specific aortic root anatomy reconstructed from computed tomography (CT) image data. These IFED simulations, which incorporate biomechanics models fit to experimental tensile test data, automatically capture the contact within the device and between the self-expanding stent and native anatomy. Further, we apply realistic driving and loading conditions based on clinical measurements of human ventricular and aortic pressures and flow rates to demonstrate that our Evolut R model supports a physiological diastolic pressure load and provides informative clinical performance predictions.

## INTRODUCTION

The prevalence of valvular heart diseases, including aortic valve stenosis, continues to increase with the average age of the world’s population.^41^ Aortic valve stenosis is a stiffening and narrowing of the valve that produces a greater resistance to blood flow and an increase in the transvalvular pressure difference. Ultimately, this condition leads to lessened blood flow to the body and increased workload for the heart, frequently resulting in substantial impacts on a patient’s quality of life. The only effective treatment currently available for severe aortic stenosis is replacement of the native valve with an artificial valve. In response to the growing number of cases of valvular disease, the annual number of valve replacements conducted world-wide is projected to grow from 300,000 in 2009 to 850,000 by 2050.^4^ Transcatheter aortic valve replacement (TAVR) introduced a less invasive alternative to surgical aortic valve replacement (SAVR) and quickly became the most common type of aortic valve replacement in the United States.^23^ Although it was originally restricted to surgically inoperable patients, in 2019 TAVR was made available to all patients who need aortic valve replacement, independent of their surgical risk.

Current TAVR devices use chemically fixated tissues, such as porcine or bovine pericardial tissue, that deteriorate over time. While SAVR is known to have a durable lifetime of only 10–15 years, the long-term durability of TAVR is still largely unknown.^34^ Varying degrees of intra- and para-valvular leakage (PVL), reduced leaflet mobility, subclinical leaflet thrombosis, calcification, pannus formation, valve tearing, and other forms of failure can occur as short- or long-term complications.^6^ Valve-in-valve TAVR, in which an additional device is implanted within a failed aortic valve replacement, has become available to address total failure of TAVR, but the ability to predict the occurrence or causes of such complications is elusive. Factors such as device sizing, rotational and translational alignment with the native valve, and patient-specific anatomy and lesion distribution may be related to these complications.

While imaging is the main diagnostic and research tool currently used to investigate such complications, common limitations, including poor resolution of the valve leaflets and of the surrounding flow structures, impede studies of these conditions. Since many of these complications are related to the fluid-structure interaction between the valve leaflets and the surrounding blood flow, computer modeling and simulation (CM&S) has the potential to aid in the process of TAVR device design, regulatory approval, and indication in patient-specific care. CM&S can assess device performance in patient-specific anatomies and under patient-specific conditions to inform individualized treatment decisions.^25^ Additionally, CM&S can isolate specific factors, allowing for root cause analyses of post-implantation complications.

Some prior computational studies of TAVR have used only finite element (FE) structural analysis to optimize device positioning by investigating the potential for post-TAVR paravalvular gaps or electrical conductivity abnormalities;^2,3,7,24,32,35,36^ however, these models do not directly address the dynamics of the valve leaflets after implantation. Other studies performed a combination of FE simulations followed by separate computational fluid dynamics (CFD) analyses to estimate PVL and assess thrombosis risk,^1,5,8,38^ but these models neglect the two-way interaction between the device and the blood. A fully coupled fluid-structure interaction (FSI) model is needed to capture the dynamics and hemodynamics of the replacement valve throughout the cardiac cycle.^42^ Several previous FSI models include partial descriptions of the TAVR device and its implantation but omit features such as the stent or interactions between the stent and the native valve.^10,11,16,21,39,40^ We are only aware of three prior studies that provide detailed descriptions of the entire TAVR device and its full interaction with the native aortic valve. Luraghi *et al*.^19,20^ present an FSI model of TAVR in a patient-specific aortic root including a calcified native valve, and Pasta *et al*.^29^ conduct an FSI study of TAVR in bicuspid aortic valve patients. However, all of these studies utilize an idealized linear elastic material model for the device’s porcine pericardial leaflets and directly apply pressure waveforms as boundary conditions, which can generate nonphysical flow oscillations during valve closure. Additionally, these models require explicit contact models to treat contact among the valve leaflets and between the TAVR device and the patient’s anatomy.

This study introduces an FSI model of TAVR with Medtronic’s *CoreValve Evolut R* device based on a hyperelastic finite element extension^13^ of Peskin’s immersed boundary (IB) method^30^ called the immersed finite element-difference (IFED) method. We build on prior FSI models of surgical bioprosthetic heart valves (BHVs) in an *in vitro* pulse-duplicator system,^17,18^ which achieved excellent agreement with experimental pressure, flow rate, and leaflet kinematic data and demonstrated that the models capture experimentally observed changes in leaflet fluttering dynamics that occur for different diameters of surgical BHVs.^18^ Herein, we simulate crimping and deployment of the Evolut R, as well as device behavior across the cardiac cycle in a patient-specific aortic root anatomy with realistic driving and loading conditions determined by reduced-order models that we fully couple to the FSI simulation. We utilize comprehensive models of the leaflet biomechanics that are fit to experimental tensile test data and allow the extraction of clinically relevant metrics, such as pre- and post-procedure transvalvular pressure differences, detailed flow patterns, leaflet dynamics, and valve orifice areas. Additionally, our IFED model intrinsically captures the contact between the self-expanding stent and the native aortic anatomy without any additional contact model. Hence, the key contribution of this study is the detailed FSI model of the TAVR procedure and device that provides clinical performance predictions by including biomechanics models fit to experimental tensile test data, applying realistic driving and loading conditions upstream and downstream through fully coupled reduced-order models, and automatically accounting for contact between the device and the patient anatomy.

## MATERIALS AND METHODS

### Anatomical and Device Geometries

**FIGURE 1.**
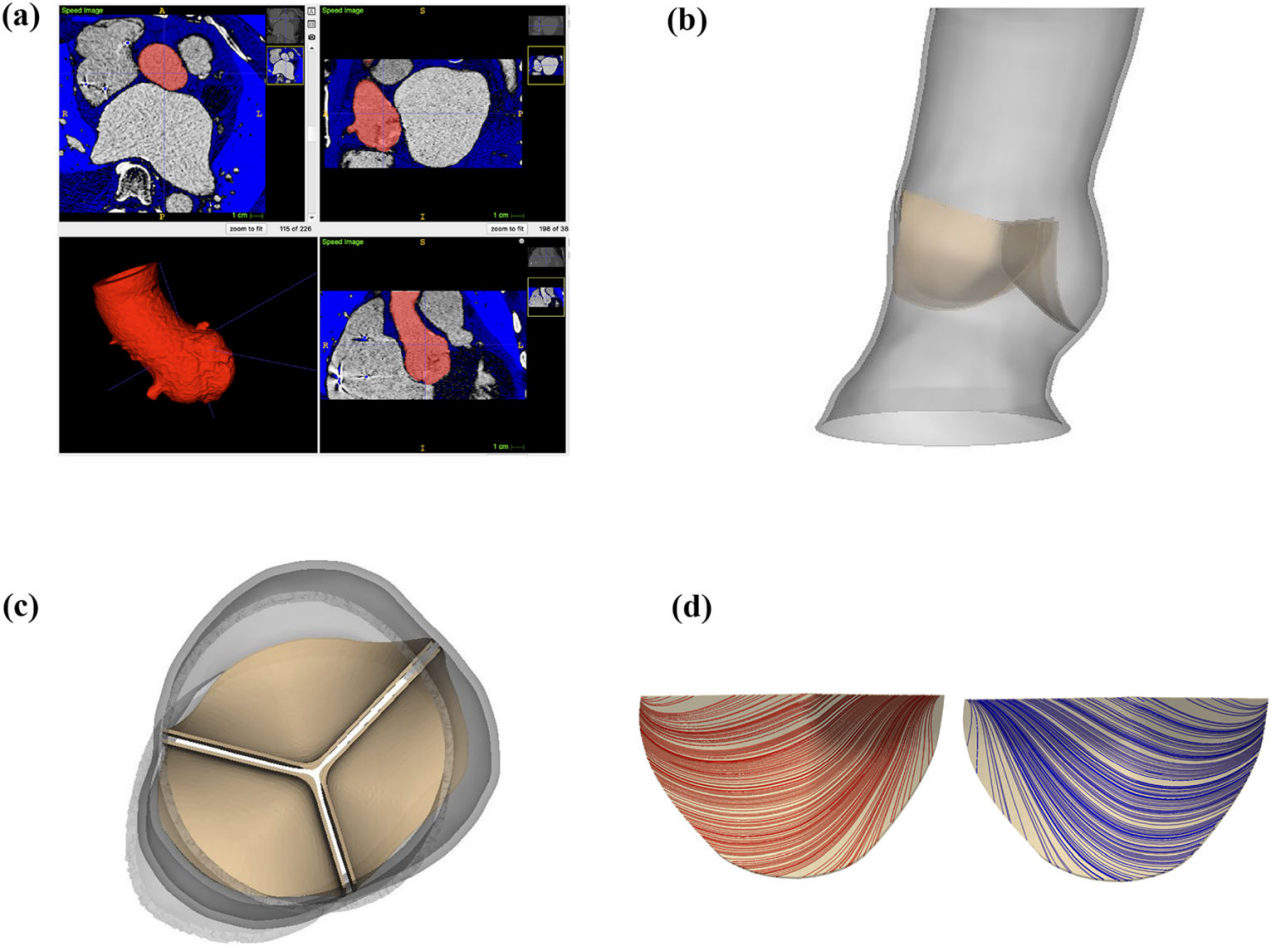
Patient-specific three-dimensional aortic root geometry from computed tomography (CT) image data. (a) Identification of patient-specific aortic root geometry through semi-automated Ct image segmentation in iTk-sNaP. (b) Reconstructed aortic root geometry. The aortic root measures 26 mm in diameter, 7.68 cm in length, and 1.0 mm in thickness. (c) Native aortic valve geometry. The idealized volumetric native aortic valve leaflets are constructed from measurements by Sahasakul *et al*.^33^ and trimmed to fit within the reconstructed aortic root. The thickness of the leaflets is 0.4 mm in the belly regions and 0.92 mm in the nodules of Arantius. (d) Native aortic valve model fiber architecture. The mean fiber orientation runs from commissure to commissure; however, two fiber directions (shown separately in red and blue) are used in each leaflet to account for fiber angle dispersion and are fit to tensile test data from Pham *et al*.^31^

We construct a three-dimensional model of a patient-specific aortic root from pre-procedural computed tomography (CT) images of a female patient selected for TAVR with a 26 mm Medtronic *CoreValve Evolut R* at UNC Medical Center (Figs. 1a and 1b). The images used in this study were obtained under UNC Institutional Review Board study number 18-0202. Since the native valve leaflets are not clearly captured in the patient’s images, we construct idealized volumetric native aortic valve leaflets (Fig. 1c) based on measurements from Sahasakul *et al.*^33^ and trim them in SOLIDWORKS (Dassault Systèmes SOLIDWORKS Corporation, Waltham, MA, USA) to fit within the reconstructed anatomy. Supplemental Materials Section A provides additional details.

**FIGURE 2.**
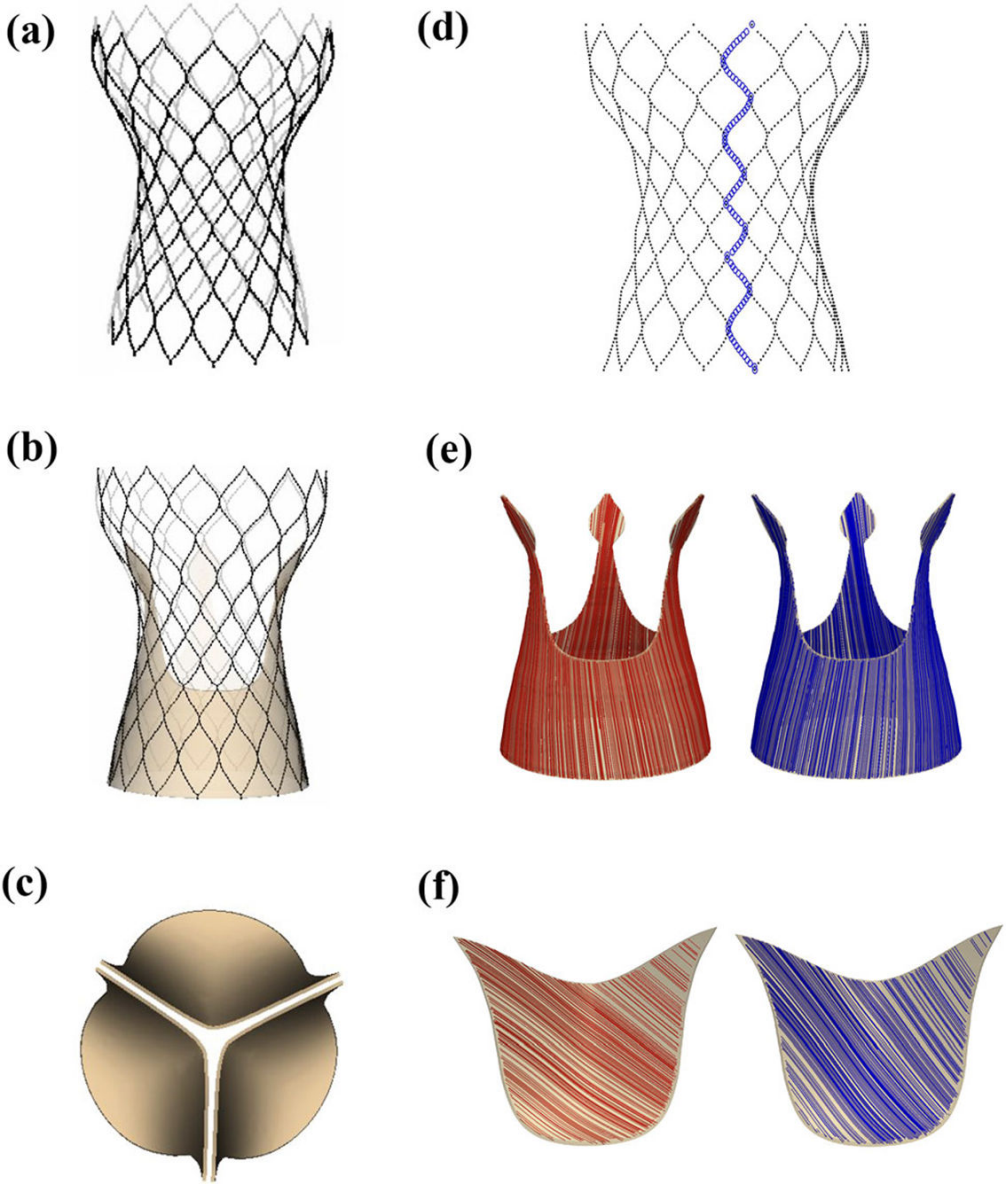
*CoreValve Evolut R* model geometry. (a) Evolut R Nitinol stent frame model geometry reconstructed from a computed tomography (CT) scan of a 26 mm Medtronic *CoreValve Evolut R* device. (b) Evolut R glutaraldehyde-fixed porcine pericardial sealing skirt geometry with a thickness of 0.34 mm. (c) Evolut R glutaraldehyde-fixed porcine pericardial leaflets geometry with a thickness of 0.34 mm. (d) Rendering of one vertical curve of stent points connected by a series of beams and springs. (e) Evolut R glutaraldehyde-fixed porcine pericardial sealing skirt fiber architecture. The mean fiber direction is 45°; however, two fiber directions (shown separately in red and blue) are used in each leaflet to account for fiber angle dispersion. (f) Evolut R glutaraldehyde-fixed porcine pericardial leaflets fiber architecture. The mean fiber direction is parallel to the direction of flow; however, two fiber directions (shown separately in red and blue) are used to account for fiber angle dispersion

Our model of the Evolut R is constructed from a CT scan of a 26 mm Medtronic *CoreValve Evolut R* device performed at UNC School of Medicine’s Biomedical Research Imaging Center. We create a discrete representation of the stent frame (Fig. 2a) by placing a series of points along the stent in the CT images. We then generate a volumetric model of the device’s porcine pericardial sealing skirt (Fig. 2b) around the stent frame and of the device’s porcine pericardial leaflets (Fig. 2c) using measurements of the physical device. Additional details are included in Supplemental Materials Section B.

### Structural Mechanics Models

#### Leaflets and Sealing Skirt

The biomechanics of the native aortic valve leaflets, the Evolut R’s glutaraldehyde-fixed porcine pericardial leaflets, and the Evolut R’s glutaraldehyde-fixed porcine pericardial sealing skirt are modeled via nonlinear solid mechanics through the use of a Lagrangian material coordinate system (Supplemental Materials Section I.1). Reference coordinates at time *t* = 0, $X=(X_{1},X_{2},X_{3})\in \Omega_{0}^{s}$, are mapped to Eulerian physical coordinates at time *t*, $x=(x_{1},x_{2},x_{3})\in \Omega_{t}^{s}$, by **x** = **χ**(**X**, *t*). We model both sets of valve leaflets and the sealing skirt as hyperelastic materials, so the first Piola-Kirchhoff stress is

(1) $$P=\frac{\partial \Psi }{\partial F},$$

in which F=∂χ∕∂X and Ψ(F) is a strain-energy functional. We use a formulation adapted from nearly incompressible elasticity and decompose Ψ(F) into its isochoric and volumetric parts:

(2) $$\Psi (F)=W(\bar{F})+U(J),$$

with J=det(F) and $\bar{F}=J^{-1∕3}F$.

For our leaflets and sealing skirt, we employ the modified Holzapfel–Gasser–Ogden (HGO) model of Murdock *et al*.^17,26^:

(3) $$W(\bar{F})=C_{10}\{\text{exp}\;[C_{01}({\bar{I}}_{1}-3)]-1\}\;\;+\frac{k_{1}}{2k_{2}}\sum_{i}\{\text{exp}\left[k_{2}({\bar{I}}_{4,i}^{⋆}-1{)}^{2}\right]-1\}.$$

Here, ${\bar{I}}_{1}=\text{tr}(\bar{C})$ is the first invariant of the modified right Cauchy–Green strain tensor $\bar{C}={\bar{F}}^{T}\bar{F}$, and ${\bar{I}}_{4,i}^{⋆}=\max({\bar{I}}_{4,i},1)=\max(f_{i}^{0}\cdot \bar{C}f_{i}^{0},1)$, in which $f_{i}^{0}$ represents a unit vector along the *i*^th^ fiber direction in the reference configuration.

We first fit the parameters for the native aortic valve leaflets to experimental planar biaxial tensile test data from Pham *et al*.^31^ for healthy human aortic valve tissue. For the native valve leaflets’ fiber directions, we assume a mean fiber direction that runs from commissure to commissure; however, the material model includes two separate families of fibers that account in a simple way for fiber angle dispersion (Fig. 1d). These fiber families are rotated within the plane of the leaflet by ±*θ* from the mean direction. Supplemental Materials Sections C and D provide additional details. Table 1 presents best-fit parameters for the healthy native aortic valve leaflets. We then create a stenotic version of the native aortic valve by increasing its isotropic stiffness, which is given by *C*_10_ in (3), by a factor of 10^3^ to create the increased resistance characteristic of severe aortic stenosis.

The parameters utilized for the glutaraldehyde-fixed porcine pericardial tissue of the Evolut R’s leaflets and sealing skirt are taken from Murdock *et al*.^26^ who fit the parameters to both planar biaxial tensile test data as well as flexural data. The mean fiber direction in the Evolut R’s pericardial leaflets is set to 45°, in accordance with the chosen orientation of the chemically fixated pericardial tissue in the construction of the device, and the mean fiber direction in the Evolut R’s pericardial sealing skirt is chosen to be parallel to the direction of flow. Two distinct fiber families (Figs. 2e and 2f) are rotated from the mean by ±*θ*. Additional details are provided in Supplemental Materials Section E, and Table 1 reports Murdock *et al*.’s parameters for glutaraldehyde-fixed porcine pericardial tissue, which are used in this study for both the Evolut R’s leaflets and sealing skirt.

The continuous formulation used in this study (Supplemental Materials Section I.1) models the structures as exactly incompressible; however, in our numerical formulation, the volumetric portion of the strain energy, *U*(*J*), acts as a stabilization term that reinforces the incompressibility constraint.^37^ We use^17^:

(4) U(J)=β[Jln(J)−J+1].

Table 1 lists the values of *β* used in this study.

#### Aortic Root

We model the vessel wall as a stiff, nearly rigid structure through the use of a penalty method [17] as detailed in Supplemental Materials Section F.

#### Stent Frame

The Evolut R’s Nitinol stent frame is modeled using a collection of Lagrangian points connected by a series of beams and springs (Fig. 2d).^14,30^ Nitinol is a nickel and titanium shape-memory alloy, which softens when the device is cooled in chilled saline during the crimping/loading process and re-hardens when warmed inside the body after deployment. The stent is self-expanding, meaning that it returns to its preferred (expanded) configuration automatically when the sheath is removed. In this initial model of the stent frame, we do not capture its temperature-dependent properties, but we do model its crimping and self-expansion as well as its contact with the native aortic valve leaflets and aortic root. Supplemental Materials Section G provides additional details.

### Fluid Model and Boundary Conditions

We model blood as a viscous incompressible fluid using the Navier-Stokes equations (Supplemental Materials Section I.1) and assign a uniform mass density *ρ* = 1.0 g cm^−3^ and dynamic viscosity *μ* = 3.5 cP. To establish realistic upstream driving and downstream loading conditions, we couple the detailed FSI model to reduced-order models^12,14^ that provide relationships between pressure and flow rate at the inlet and outlet of the FSI model.

A three-element Windkessel model establishes downstream loading conditions:

(5) $$C\frac{dP_{\text{Wk}}}{dt}=Q_{\text{Ao}}-\frac{P_{\text{Wk}}}{R_{p}},$$

(6) $$P_{\text{Ao}}=P_{\text{Wk}}+Q_{\text{Ao}}R_{c},$$

in which *C* is the compliance, *R*_c_ is the characteristic resistance, *R*_p_ is the peripheral resistance, *P*_Wk_ is the Windkessel pressure, and *Q*_Ao_ and *P*_Ao_ are the volumetric flow rate and mean pressure at the outlet of the FSI model. For the upstream driving conditions, we employ a time-dependent elastance-based left heart model:

(7) $$\frac{d(C_{\text{LA}}\;P_{\text{LA}})}{dt}=Q_{\text{vein}}-Q_{\text{MV}},$$

(8) $$\frac{d(C_{\text{LV}}\;P_{\text{LV}})}{dt}=Q_{\text{MV}}-Q_{\text{LVOT}},$$

(9) $$P_{\text{LVOT}}=P_{\text{LV}}-Q_{\text{LVOT}}R_{\text{LVOT}},$$

(10) QMV={0,PLA≤PLV,PLA−PLVRMV,PLA>PLV,}

in which *C*_LA_ and *C*_LV_ are the time-dependent compliances of the left atrium (LA) and left ventricle (LV), *R*_LVOT_ and *R*_MV_ are the resistances of the left ventricular outflow tract (LVOT) and mitral valve (MV), which is modeled as a diode, *P*_LA_ and *P*_LV_ are the left atrial and ventricular pressures, and *Q*_vein_, *Q*_MV_, and *Q*_LVOT_ are the volumetric flow rates of the pulmonary vein, MV, and LVOT. In this model, *Q*_vein_ is prescribed as a constant inflow rate into the LA, and the state of the MV model (open or closed) is lagged for simplification. For the parameterization of *C*_LA_(*t*) and *C*_LV_(*t*), we utilize the “two-Hill” function waveform for elastance *E*(*t*) = 1.0/*C*(*t*)^28^:

(11) $$E(t)=k\left(\frac{g_{1}}{1+g_{1}}\right)\left(\frac{1}{1+g_{2}}\right)+E_{\text{min}},$$

(12) $$g_{1}={\left(\frac{t}{\tau_{1}}\right)}^{m_{1}},\;\;g_{2}={\left(\frac{t}{\tau_{2}}\right)}^{m_{2}},$$

(13) $$k=\frac{E_{\max}-E_{\text{min}}}{\max\left[\left(\frac{g_{1}}{1+g_{1}}\right)\left(\frac{1}{1+g_{2}}\right)\right]}.$$

The values of the parameters used for the upstream model are *Q*_vein_ = 5.8 L min^−1^, *R*_MV_ = 0.005 mmHgmL^−1^ s, and *R*_LVOT_ = 0.0043 mmHgmL^−1^ s. The period of the cardiac cycle is *T* = 0.8512s. Then, for the elastance waveform of the LA, *τ*_1_ = 0.1150 · *T*, *τ*_2_ = 0.1882 · *T*, *m*_1_ = 1.32, *m*_2_ = 13.1, *E*_min_ = 0.08 mmHgmL^−1^, and *E*_max_ = 0.17 mmHgmL^−1^. Additionally, because of the timing of LA contraction in comparison to LV contraction, this LA elastance waveform is shifted by 0.85 · *T* relative to the LV elastance waveform. For the elastance waveform of the LV, *τ*_1_ = 0.0725 · *T*, *τ*_2_ = 0.4503 · *T*, *m*_1_ = 2.7463, *m*_2_ = 21.5683, *E*_min_ = 0.01 mmHgmL^−1^, and *E*_max_ = 0.1191 mmHgmL^−1^. Then, the downstream Windkessel model parameters are *R*_c_ = 0.042 mmHgmL^−1^ s, *R*_p_ = 0.9046 mmHgmL^−1^ s, and *C* = 1.9504 mLmmHg^−1^. These parameters are fit to experimental measurements of human ventricular and aortic pressures and flow rates from Murgo *et al*.^27^ for a “Type A” beat. Supplemental Materials Section H provides additional details.

### Fluid–Structure Interaction

This model utilizes the IFED method^13^ to simulate the fluid-structure interaction of the Evolut R, native aortic valve, and aortic root with the surrounding blood. Supplemental Materials Section I provides details on the continuum formulation and numerical approximations.

### Simulations

We perform dynamic simulations of crimping of the Evolut R, expansion of the Evolut R into the patient-specific aortic root with the stenotic native valve model, and the implanted Evolut R throughout the cardiac cycle. Additionally, we simulate the healthy native valve throughout the cardiac cycle to tune our boundary condition model; results for the healthy native valve are included in Supplemental Materials Section H.

#### Crimping the Evolut R

**FIGURE 3.**
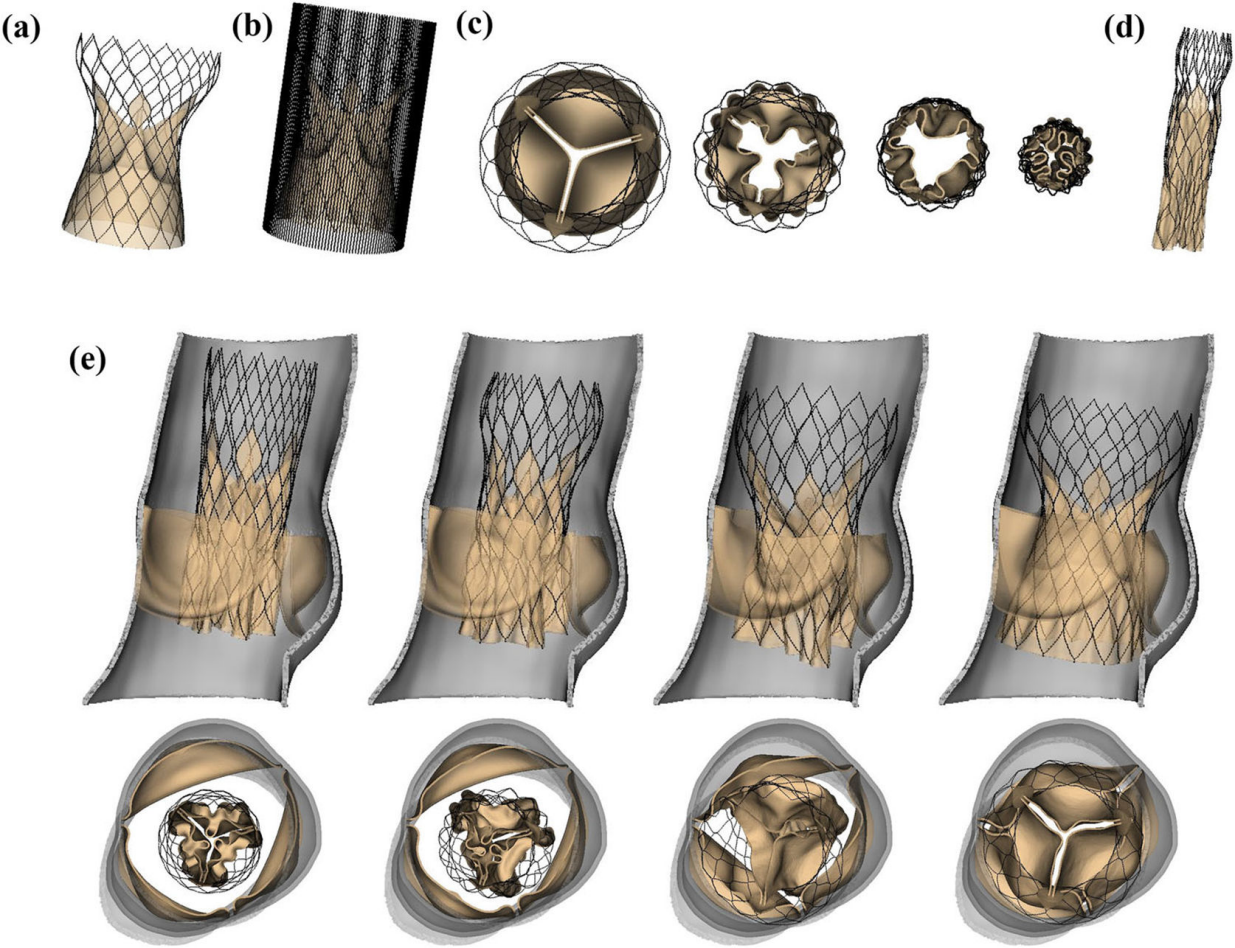
Evolut R crimping and deployment. (a) Side view of the uncrimped Evolut R model. (b) Side view of the Evolut R with model crimping device. (c) Top views of the crimping process. The model crimping device applies radial force to the stent frame to crimp the Evolut R. Contact is handled implicitly via the immersed finite element-difference (IFED) method. (d) Side view of the crimped Evolut R model. The final crimped diameter is approximately 17.25 Fr. (e) Expansion of the Evolut R within the stenotic native valve. The top row gives a cross-sectional view of the native valve and Evolut R model kinematics during expansion, and the bottom row provides a top view of the valve at coinciding times

We construct a model crimping device (Fig. 3b) from material points, similar to the construction of the stent frame described in Supplemental Materials Section G. The vertical curves of the crimping cylinder are also connected by beams and springs with the same beam and spring constants as the stent. To crimp the Evolut R, the points in the crimping device are tethered with a stiff spring constant of 3.0 × 10^8^ dyne/cm to target points, which move inward radially at a constant velocity of 50.0 cm/s, uniformly compressing the stent (Fig. 3c). Contact between the crimping device and the stent frame and between the stent frame and the porcine pericardial tissue that composes the Evolut R’s leaflets and sealing skirt is handled automatically by the IFED method.

#### Deploying the Evolut R

To deploy the device, we fully open the stenotic native valve by applying a pressure of 750 mmHg to the underside of the valve leaflets. After the native valve opens, we release the Evolut R from its crimped configuration, allowing it to self expand inside the native valve under the restoring forces from the network of beams and springs that compose the stent frame, as described in Supplemental Materials Section G.

#### The Evolut R Across the Cardiac Cycle

Finally, we perform dynamic simulations of the implanted Evolut R across the cardiac cycle using the boundary conditions described in the “Fluid Model and Boundary Conditions” section.

## RESULTS

We present data output by our model in the three situations described in the “Simulations” section. Figures 3a-3d depict crimping of the Evolut R model. The final crimped diameter (Fig. 3d) is approximately 17.25 Fr, which is appropriate for the 18 Fr valve capsule used clinically to deliver the device to the native valve.^22^

Figure 3e illustrates the self-expansion of the Evolut R within the stenotic native valve. The stent frame contacts the wall of the aortic root as well as the underside of the native valve but does not penetrate either as a consequence of the IFED method’s implicit contact model. We also confirm that the final position of the device within the native aortic root meets the clinical guidelines for optimal placement, with the porcine pericardial sealing skirt 4–6 mm below the aortic annulus, which is in the optimal implant zone just above the second radiopaque band of the stent.^22^

**FIGURE 4.**
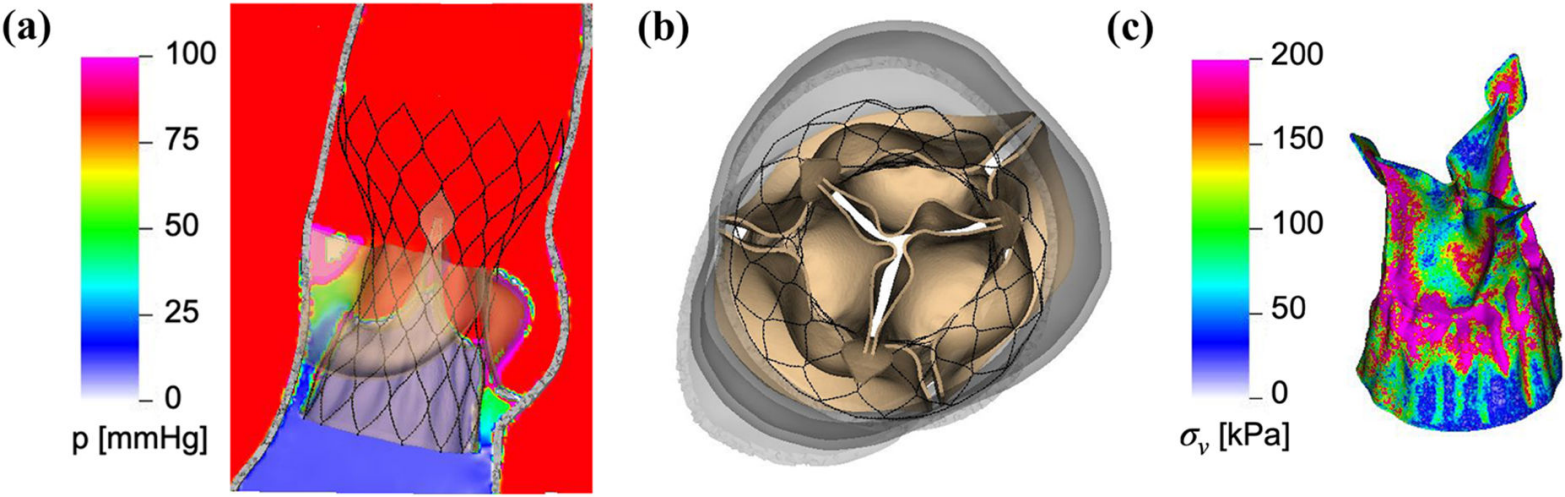
Diastolic pressure loading of the deployed Evolut R, shown at the time of peak transvalvular pressure difference. (a) Cross-sectional view of detailed diastolic pressure pattern for the deployed Evolut R model at the time of peak transvalvular pressure difference. The color indicates the pressure at the center plane of the valve, with cooler colors indicating lower pressures and warmer colors indicating higher pressures. (b) Top view of the pressure-loaded Evolut R model at the time of peak transvalvular pressure difference. The transvalvular pressure difference at this time is approximately 72 mmHg, but the Evolut R remains in place within the stenotic native aortic valve. (c) Detailed von Mises stress distribution for the porcine pericardial leaflets and sealing skirt of the deployed Evolut R model at the time of peak transvalvular pressure difference. The color indicates the von Mises stress, with cooler colors indicating lower stresses and warmer colors indicating higher stresses

After expansion, we pressurize the deployed Evolut R with a physiological diastolic pressure load. Figure 4 depicts the fully pressure-loaded deployed Evolut R at the time of peak transvalvular pressure difference, which is 72 mmHg. The forces produced by the beams and springs that push the stent frame to its fully expanded configuration, combined with the implicit contact model of the IFED method, keep the Evolut R in place within the native aortic valve under the diastolic pressure load.

**FIGURE 5.**
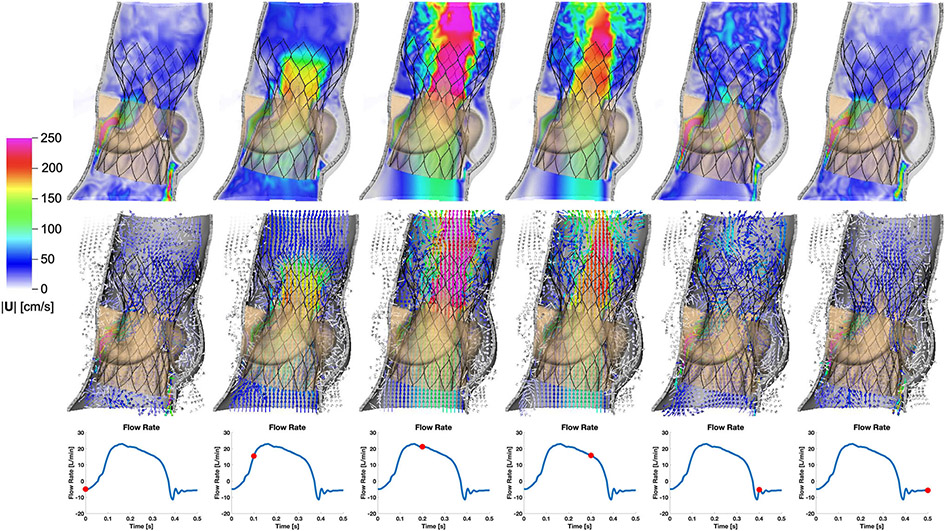
Cross-sectional view of detailed flow patterns for the Evolut R model using driving conditions based on the time-dependent elastance-based left heart model, as given by (7), (8), (9), and (10). The color indicates the magnitude of the velocity through the aortic root at the center plane of the valve, with cooler colors indicating lower velocities and warmer colors indicating higher velocities. The time increment between frames is 0.1 s. The first row shows the velocity magnitude at each location in the plane, while the second row displays a sampling of normalized velocity vectors

**FIGURE 6.**
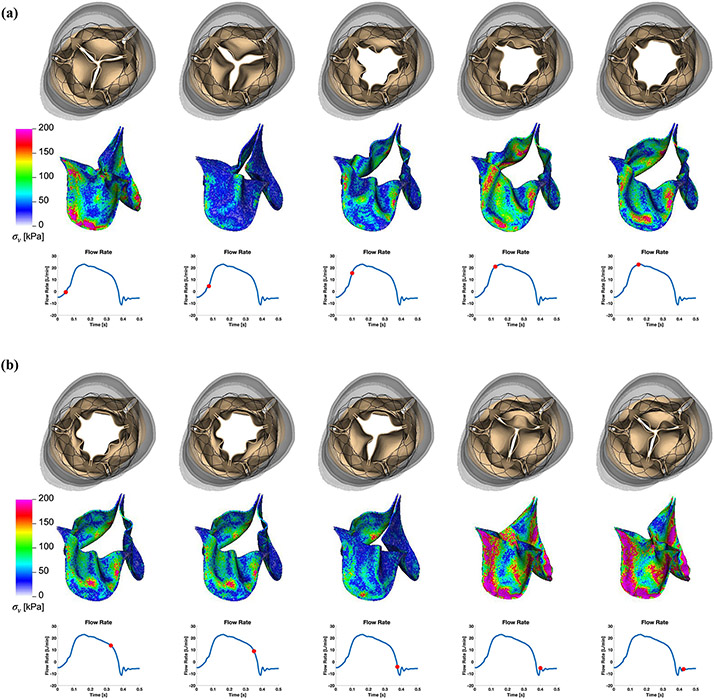
(a) Leaflet kinematics and von Mises stress distributions of the Evolut R model during valve opening. The color in the second row indicates the von Mises stress, with cooler colors indicating lower stresses and warmer colors indicating higher stresses. The time increment between frames is 25 ms. (b) Leaflet kinematics and von Mises stress distributions of the Evolut R model during valve closing. The color in the second row indicates the von Mises stress, with cooler colors indicating lower stresses and warmer colors indicating higher stresses. The time increment between frames is 25 ms

**FIGURE 7.**
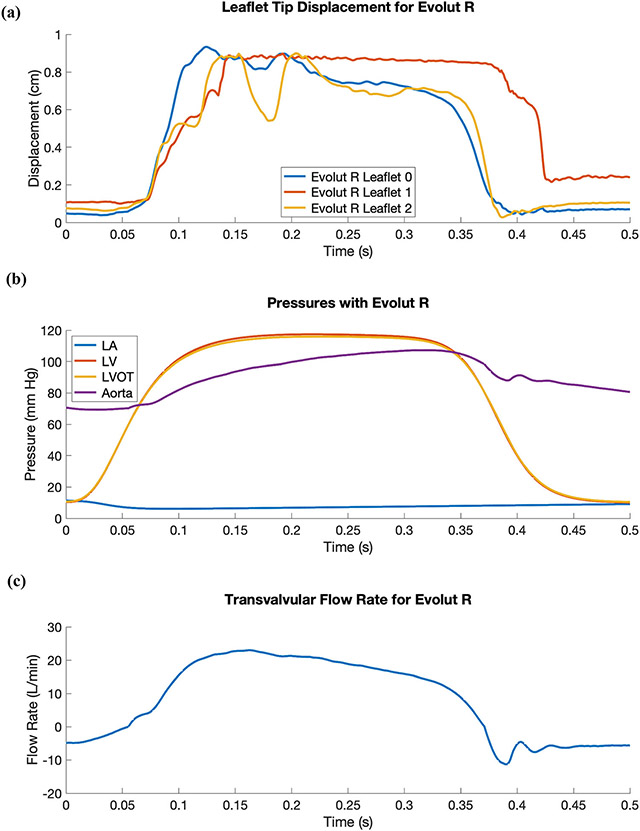
Leaflet tip displacement, pressure, and flow rate waveforms for the Evolut R model using driving conditions based on the time-dependent elastance-based left heart model, as given by (7), (8), (9), and (10). (a) Leaflet tip displacement during systole for the Evolut R’s porcine pericardial leaflets. (b) Left atrial (LA), left ventricular (LV), left ventricular outflow tract (LVOT), and aortic pressure waveforms with the implanted Evolut R. The mean systolic transvalvular pressure difference is 9.62 mmHg, and the effective orifice area (EOA) is 1.50 cm^2^. (c) Transvalvular flow rate for the implanted Evolut R. The stroke volume is 84.27 mL, yielding a cardiac output of 5.91 L/min

Finally, we perform dynamic simulations of the Evolut R across the cardiac cycle. Figure 5 displays detailed flow patterns within and downstream of the Evolut R, which continues to remain implanted within the stenotic native valve, despite the intense flow jet that passes through the valve leaflets during systole. Figure 6 depicts leaflet kinematics and von Mises stress distributions of the Evolut R during valve opening (Fig. 6a) and closing (Fig. 6b). Figure 7a plots the displacements of the Evolut R leaflets’ tips from the center of the valve to track fluttering during systole. Figure 7 also plots the pressure (Fig. 7b) and flow rate (Fig. 7c) waveforms for the Evolut R model using the elastance-based left heart model described in the “Fluid Model and Boundary Conditions” section. Under these boundary conditions, the mean systolic transvalvular pressure difference is 9.62 mmHg, and the stroke volume is 84.27 mL, yielding a cardiac output of 5.91 L/min. Additionally, we use the simplified continuity equation to compute the effective orifice area (EOA) of the Evolut R, which is 1.50 cm^2^. These pressure difference and EOA values are in good agreement with clinical data measured from patients with 26 mm Evolut Rs by Hahn *et al*.^15^ who report a mean pressure difference of 7.53 ± 2.65 mmHg and an EOA of 1.69 ± 0.40 cm^2^.

**FIGURE 8.**
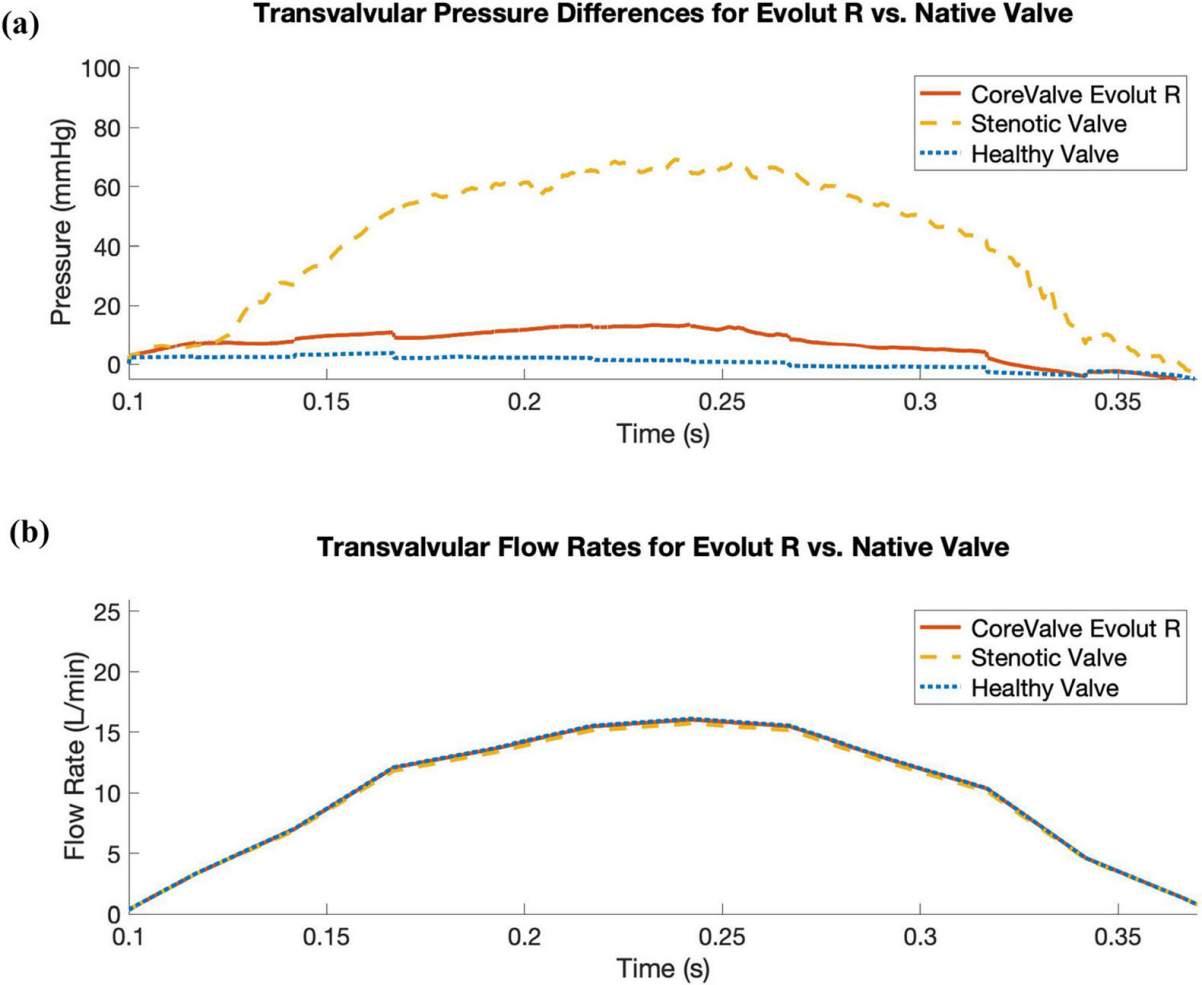
Comparison of systolic transvalvular pressure difference waveforms for the Evolut R model, the stenotic native valve model, and the healthy native valve model, while prescribing a flow rate waveform measured by Garcia *et al*.^9^ for a patient with severe aortic valve stenosis. (a) Simulated systolic transvalvular pressure differences for the Evolut R, the stenotic native valve, and the healthy native valve. The mean systolic transvalvular pressure differences in the simulated results are: 6.84 mmHg for the Evolut R, 41.42 mmHg for the stenotic native valve, and 0.47 mmHg for the healthy native valve. (b) Prescribed transvalvular flow rate measured by Garcia *et al*. for a patient with severe aortic valve stenosis. The stroke volume is 45.32 mL, yielding a cardiac output of 3.47 L/min

To asses the impact of the valve replacement procedure, we characterize the valvular flow resistance pre- and post-TAVR with the mean transvalvular pressure difference and EOA, while applying a common systolic flow rate waveform for direct comparison. We prescribe the flow waveform (Fig. 8b) measured by Garcia *et al*.^9^ from “Patient 2,” who had severe aortic valve stenosis and was awaiting valve replacement. Figure 8a compares the transvalvular pressure difference waveforms between the Evolut R, the stenotic native valve, and the healthy native valve models, and Table 2 compares the mean pressure differences and EOAs. Under these boundary conditions, the mean pressure differences for the healthy native valve model, stenotic native valve model, and Evolut R TAVR model are 0.472 mmHg, 41.42 mmHg, and 6.84 mmHg, respectively. Additionally, the EOAs for the healthy native valve model, stenotic native valve model, and Evolut R TAVR model are 3.63 cm^2^, 0.76 cm^2^, and 1.32 cm^2^, respectively. Table 2 also demonstrates the consistency between the pressure difference and EOA values for the Evolut R model and the clinical data from Hahn *et al*.^15^ for valves of the same type and size, with a mean pressure difference of 7.53 ± 2.65 mmHg and an EOA of 1.69 ± 0.40 cm^2^.

## DISCUSSION

This study presents a dynamic computational fluid-structure interaction model of transcatheter aortic valve replacement using the immersed finite element-difference method. We model Medtronic’s *CoreValve Evolut R* TAVR device, which we crimp and deploy in a clinical image-based, patient-specific aortic root geometry with a stenotic native aortic valve and simulate across the cardiac cycle. The model includes anisotropic descriptions of both the native aortic valve leaflets and the glutaraldehyde-fixed porcine pericardial tissue that composes the Evolut R’s leaflets and sealing skirt. These detailed material models are fit to experimental tensile and flexural test data from Pham *et al*.^31^ and Murdock *et al*.^26^ and allow for assessment of clinically relevant performance metrics that are not available from standard imaging techniques, such as leaflet fluttering patterns (Fig. 7a) and von Mises stress distributions (Fig. 6), the latter of which cannot be readily measured *in vivo* or *in vitro*. In this case, the leaflet fluttering shown in Figure 7a is minimal, which we have hypothesized to be a positive factor for long-term durability.^18^ The von Mises stresses on the leaflets depicted in Figure 6 are distributed according to the asymmetric fiber orientation in the porcine pericardial tissue and the interaction of the device with the stent and surrounding native valve. During opening (Fig. 6a), the von Mises stresses are concentrated at locations on the outer rim of the leaflet that directly interact with the flow jet without support from the surrounding native valve. During closing and diastolic loading (Figs. 4c and 6b), the highest leaflet stresses occur at points of attachment to the stent frame. The relatively high stresses are consistent with those previously observed in porcine valves and may be related to higher rates of tearing in porcine BHVs.^17^

Additionally, the model includes a self-expanding model of the Evolut R’s stent frame, the geometry of which is reconstructed from CT image data. Since we are able to fully crimp our Evolut R model (Fig. 3d) to a diameter of 17.25 Fr, even with our volumetric porcine pericardial leaflets and sealing skirt, the framework could be expanded to investigate the hypothesis that stent-crimp induced injury to TAV leaflets can induce leaflet thrombosis.^6,34^ We also employ physiological driving and loading conditions using reduced-order models that are based on clinical measurements from healthy human subjects,^27^ including an upstream time-dependent elastance-based left heart model and a downstream Windkessel model. The upstream and downstream models are independently calibrated before being fully coupled to the FSI model. Simulated pressures, flow rates, and kinematics result from the integration of the three models and are not prescribed. The main effect of the boundary condition model is to establish a pressure and flow relationship for the boundaries, whereas directly prescribing either a pressure or flow rate waveform would not allow the model to capture the effects of both diastolic pressure loading and valvular flow resistance.

Many features of our Evolut R model are enhanced by the implicit contact model provided by the IFED method as a consequence of the fact that all structural models move according to a common background velocity field for both the fluid and solid regions that is continuous at the fluid-structure interfaces. Successful crimping of the device using a model crimper, implantation into the aortic root within the native valve, and contact among the stent frame, aortic wall, native leaflets, Evolut R leaflets, and Evolut R sealing skirt throughout the cardiac cycle all rely on contact between dynamic structures that are also interacting with the surrounding fluid. After implantation, the forces generated by the network of beams and springs that compose the stent frame hold it in its expanded position against the wall of the aortic root and the native aortic valve. Figure 4 shows that the model supports a realistic pressure load during diastole, both in the sense that the Evolut R remains secure within the aortic root, and that the closed Evolut R leaflets form a seal without intravalvular leaks. The “gap” between the valve leaflets when the valve is closed reflects the width of the regularized delta function that is used to couple Lagrangian and Eulerian variables in the IFED method (Supplemental Materials Section I.2); however, the valve is indeed closed with respect to the fluid. The model’s ability to support the physiological diastolic pressure load imposed by the reduced-order boundary condition models is also important to assess paravalvular leak, a common post-implantation complication. The final frame of Fig. 5 demonstrates the model’s ability to capture the small-scale flow features of a slight paravalvular leak, which is visible at the bottom right of the sealing skirt. Additionally, during systole, the Evolut R is not embolized by the high-velocity flow jet that passes through its open leaflets, as shown in Figs. 5 and 6.

Our model produces pre- and post-TAVR results consistent with those seen clinically,^15^ in regards to mean pressure difference and EOA, as demonstrated in Table 2. The stenotic native valve model is considered severely stenotic both because the mean pressure difference exceeds 40 mmHg and because the EOA is less than 1.0 cm^2^. Additionally, the post-operative mean pressure gradient and EOA are consistent with clinical data for this device.^15^ However, there are several limitations of the current model. Although we assume a nearly rigid model of the aortic root to simplify the application of boundary conditions, in reality, the aorta has significant compliance. Future work will involve capturing this compliance in the model description, which we anticipate will also increase agreement between simulated and experimental pressures and flow rates. We also plan to further develop our reduced-order boundary models to better replicate experimental data. Further, we are not currently capturing the mechanical properties of Nitinol in our model of the stent frame. Although our current approach is likely sufficient for our focus on the implanted Evolut R in its expanded configuration, future work will involve incorporating a description of Nitinol and making our model of the device deployment procedure more realistic with the addition of a sheath that can be gradually removed from the stent frame, rather than releasing the entire stent for expansion simultaneously. Additionally, while the numerical method used in this study cannot resolve wall shear stresses, sharp interface methods are being developed to resolve the boundary layer at fluid-solid interfaces. Finally, this initial model has not yet been substantially validated. Future work will include *in vitro* experiments for rigorous comparison and validation via particle image velocimetry, leaflet dynamics comparisons, and pressure and flow data. Additional patient-specific modeling will also be conducted in cases with pre- and post-operative data available for detailed comparison to clinical outcomes.

Overall, this study introduces an effective computational model framework for fluid-structure interaction of TAVR devices within patient-specific anatomies, in which device behavior and hemodynamics is simulated across the cardiac cycle. Computational modeling and simulation (CM&S) of TAVR devices can allow researchers to assess device performance and durability under a broader range of conditions than traditional *in vitro* experiments and can ultimately be used to address persistent challenges encountered by TAVR device designs and to investigate and predict post-implantation complications. Additionally, these models could be extended to study the use of TAVR devices in bicuspid aortic valves and in valve-in-valve TAVR procedures. Ultimately, CM&S of TAVR devices with patient-specific models has the potential to allow clinicians to individualize treatment decisions based on thorough computational investigation of post-implantation performance, thus improving patient outcomes.

## Supplementary Material

Supplementary Materials

Video #1

Video #2

Video #3

Video #4

## Figures and Tables

**TABLE 1.** HGO constitutive model & volumetric stabilization parameters for the healthy native valve leaflets & glutaraldehyde-fixed porcine pericardial tissue.

	*C*_10_ [kPa]	*C*_01_	*k*_1_ [MPa]	*k*_2_	*θ* [deg.]	*β* [MPa]
Healthy native valve leaflets	0.1463	26.21	0.007072	147.5	26.21	141.0
Porcine pericardial tissue	15.14	13.48	0.1526	107.3	7.81	5.840

Parameters for the fiber-reinforced HGO model described by (3) as well as the volumetric stabilization energy given in (4) for the healthy native aortic valve leaflets and for the glutaraldehyde-fixed porcine pericardial tissue that composes the Evolut R’s leaflets and sealing skirt. The mean fiber direction for the native aortic valve leaflets is commissure-to-commissure, whereas the mean direction for the Evolut R’s glutaraldehyde-fixed porcine pericardial leaflets is 45°. The mean fiber direction for the Evolut R’s glutaraldehyde-fixed porcine pericardial sealing skirt is parallel to the direction of flow. The native aortic valve leaflet parameters are fit to data from Pham *et al*. [31], whereas the glutaraldehyde-fixed porcine pericardial tissue parameters originate from Murdock *et al*. [26].

**TABLE 2.** Mean systolic transvalvular pressure differences and effective orifice areas (EOAs) pre- and post-TAVR.

	Mean pressure Δ (mmHg)	EOA (cm^2^)
Healthy native valve model	0.472	3.63
Stenotic native valve model	41.42	0.76
Evolut R TAVR model	6.84	1.32
Evolut R clinical data[15]	7.53 ± 2.65	1.69 ± 0.4

Mean systolic transvalvular pressure differences and effective orifice areas (EOAs) from our pre- and post-TAVR models. To allow for direct comparison, we apply the flow rate waveform measured by Garcia *et al*.^9^ from a patient with severe aortic valve stenosis in each simulated case. The final row shows clinical data from Hahn *et al*.^15^ for patients that received the 26 mm Evolut R valve that we model in this study.
