# Supplementary material for: Patient–Specific Immersed Finite Element–Difference Model of Transcatheter Aortic Valve Replacement
Source: Ann Biomed Eng. Author manuscript; Available in PMC 2023 Jan 14. (PMC9832092; doi:10.1007/s10439-022-03047-3)
Supplement: Supplementary Materials [file NIHMS1849976-supplement-Supplementary_Materials.pdf]

Supplemental Materials

A Reconstruction of the Patient-specific Aortic Root Geometry

We construct a three-dimensional model of a patient-specific aortic root from pre-procedural CT image data of a female patient selected for TAVR with a 26 mm Medtronic *CoreValve Evolut R* at UNC Medical Center, as described in the "Anatomical and Device Geometries" section of the main text. The images used in this study were obtained under a protocol approved by the UNC Institutional Review Board (study number 18-0202). The CT scan was performed using a Siemens SOMATOM Definition CT Scanner with an image resolution of $512 \times 512 \times 226$ and a voxel size of $0.44140625 \times 0.44140625 \times 0.6$ mm. Segmentation of the CT images (Fig. 1a of the main text) is by a semi-automated method in ITK-SNAP,[21] which implements an active contour model that minimizes an energy functional of voxel intensities. The aortic root (Fig. 1b of the main text) measures 26 mm in diameter, 7.68 cm in length, and 1.0 mm in thickness. The inflow boundary of the model is truncated at the LVOT, and the outflow boundary of the model is truncated downstream of the aortic valve before the first arterial bifurcation. Artificial circular extensions are added at both boundaries using SOLIDWORKS to simplify the application of boundary conditions to the computational model. The radius of the vessel at both truncations is 21 mm. Since the native valve leaflets are not captured clearly enough in the CT images for precise reconstruction, we construct idealized volumetric native aortic valve leaflets (Fig. 1c of the main text) based on the measurements from Sahasakul et al.,[17] use the limited visibility of the leaflets in the CT images to accurately position the leaflets in the reconstructed aortic root, and trim them to fit within the geometry in SOLIDWORKS. The thickness of the leaflets is 0.4 mm in the belly regions and 0.92 mm in the nodules of Arantius.[1]

B Construction of the *CoreValve Evolut R* Geometry

Our model of the Evolut R is constructed from a CT scan of a 26 mm Medtronic *CoreValve Evolut R* device performed at UNC School of Medicine's Biomedical Research Imaging Center as described in the "Anatomical and Device Geometries" section of the main text. We create a discrete representation of the stent frame (Fig. 2a of the main text) using the CT images to manually place points along the stent's 30 vertical curves with a spacing of approximately 0.5 mm. We then create a model of the device's porcine pericardial sealing skirt (Fig. 2b of the main text) in SOLIDWORKS by knitting a surface around the stent frame with circular horizontal guiding curves. We shape and trim the skirt to measurements of the physical device and extrude the surface radially to reproduce the three-dimensional geometry with a thickness of 0.34 mm. Lastly, we construct an idealized volumetric model of the device's porcine pericardial leaflets (Fig. 2c of the main text) using a combination of MATLAB (The MathWorks, Inc., Natick, MA, USA) and SOLIDWORKS. We assume a uniform geometry across the three leaflets and parameterize the seam and free edge curves of the leaflet geometry, while imposing measurements of the physical valve, such as the leaflet

diameter: 27 mm, commissure height: 17 mm, and central height of the free edge: 10.625 mm. We then create a spanning surface representation of the leaflet by sweeping between the two outline curves with a series of parametric curves, which we convert into a surface mesh in MATLAB. To create the final volumetric version of the leaflet, we thicken this surface representation uniformly in SOLIDWORKS to a thickness of 0.34 mm.

C Parameter Fitting for the Native Aortic Valve Material Model

The parameters for the native aortic valve leaflets are fit (Fig. S1) to experimental planar biaxial tensile test data from Pham et al.,[16] as mentioned in the "Leaflets and Sealing Skirt" section of the main text. These tests involved mounting the human tissue specimens onto a device such that the circumferential and radial directions were aligned with the applied forces in the stress-controlled protocol. This orientation coincides with the commissure-to-commissure orientation of the collagen fibers in the native aortic valve leaflets. To fit the constitutive model parameters, we compare the second Piola-Kirchhoff stress generated by the model to the experimental values of the stress for the given values of the Green-Lagrange strain $\mathbb{E} = (\mathbb{C} - \mathbb{I})/2$. The second Piola-Kirchhoff stress is computed as follows:

$$\mathbb{S} = \mathbb{S}^{\text{dev}} - p\mathbb{C}^{-1}, \tag{S1}$$

$$\mathbb{S}^{\text{dev}} = 2\frac{\partial W}{\partial \mathbb{C}}, \tag{S2}$$

$$p = \frac{\mathbb{S}^{\text{dev}}_{33}}{(\mathbb{C}^{-1})_{33}}, \tag{S3}$$

with W described by (3) in the main text, $\mathbb{S}^{\text{dev}}$ indicating the deviatoric portion of $\mathbb{S}$, and p being the pressure under the assumption that the tissue specimens only undergo in-plane loads.

For the native aortic valve leaflets' fiber directions, we assume a mean fiber direction that runs from commissure to commissure, but we use two separate families of fibers (Fig. 1d in the main text), which are rotated within the plane of the leaflet by $\pm\theta$ from the mean direction, to account for fiber angle dispersion. Here, θ is an additional angular parameter that we also fit using the data from Pham et al., since fiber angle dispersion is needed to obtain a high-quality fit to the available tensile test data. We utilize MATLAB's `lsqcurvefit`, a nonlinear least-squares optimizer, to calculate the best-fit parameters for the native aortic valve leaflets. These parameters are presented in Table 1 in the main text.

D Fiber Structure Generation for the Native Aortic Valve Model

For the native aortic valve leaflets' fiber directions, we assume a mean fiber direction that runs from commissure to commissure, but we use two separate families of fibers (Fig. 1d in the main text), which are rotated within the plane of the leaflet by $\pm\theta$ from the mean

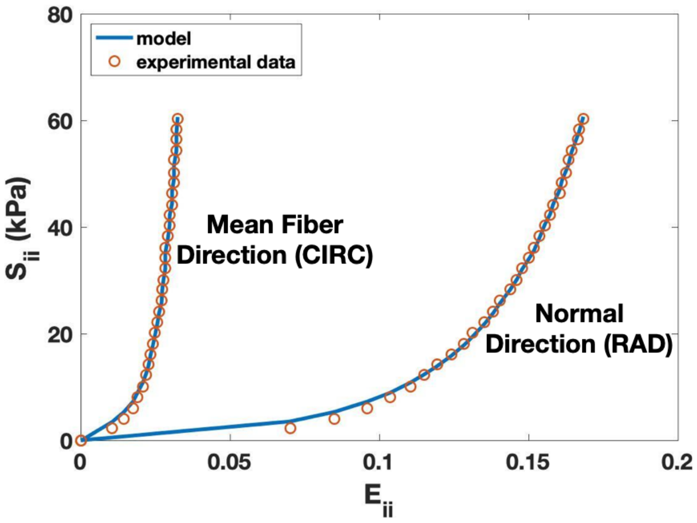

Figure S1: Parameter fitting for the native aortic valve using the experimental planar biaxial tensile test data from Pham et al. These tests involved mounting the human tissue specimens onto a device such that the circumferential and radial directions were aligned with the applied forces in the stress-controlled protocol. This orientation coincides with the commissure-to-commissure mean orientation of the collagen fibers in the native aortic valve leaflets. The best-fit parameters for the native aortic valve leaflets to this stress-strain data from Pham et al. are presented in Table 1 in the main text.

direction, to account for fiber angle dispersion. The native valve model's fiber structure is generated using a rule-based technique based on Poisson interpolation.[20] As described in detail by Hasan et al.,[7] we construct a local material coordinate system at each point in the leaflet by solving a series of Poisson problems on the leaflet geometry with different boundary conditions. By combining the gradients of the resulting scalar fields, we obtain a right-handed orthogonal triad that includes the circumferential (commissure-to-commissure) direction along the leaflet, its normal (radial) direction along the leaflet, and the out-of-plane surface normal to the leaflet. Thus, by rotating the circumferential direction vector about the surface normal vector by $\pm\theta$, we can construct the two fiber directions used in the model (Fig. 1d in the main text).

E Fiber Structure Generation for the *CoreValve Evolut R* Valve and Skirt Models

The mean fiber direction in the Evolut R's pericardial leaflets is set to 45°, in accordance with the chosen orientation of the chemically fixated pericardial tissue in the construction of the device, and the mean fiber direction in the Evolut R's pericardial sealing skirt is chosen to be parallel to the direction of flow. However, as before, two distinct fiber directions (Figs. 2e and 2f in the main text) that are rotated from the mean by $\pm\theta$ are used in the model of each structure to account for fiber angle dispersion. The fiber structure of each model is then generated using an adaptation of the rule-based technique described previously

in Supplemental Materials Section D. For the pericardial leaflets, a bisecting plane for each leaflet is used in combination with the normal vector to the leaflet surface produced by Poisson interpolation to compute the vector aligned with the 45° mean fiber direction. For the skirt, the centerline vector is used in combination with the normal vector to the skirt surface to compute the vector aligned with the mean fiber direction, which is parallel to the direction of flow.

F Aortic Root Material Model

We model the vessel wall as a stiff, nearly rigid structure through the use of a penalty method[12] that describes the aortic root as a neo-Hookean material with the energy

$$W_{\text{root}} = \frac{c_{\text{root}}}{2}(\bar{I}_1 - 3) \tag{S4}$$

and additional structural forces given by

$$\boldsymbol{F}(\boldsymbol{X}, t) = \kappa_{\text{root}}(\boldsymbol{X} - \boldsymbol{\chi}(\boldsymbol{X}, t)). \tag{S5}$$

In the limit as $c_{\text{root}} \to \infty$, the aortic root becomes perfectly rigid, and as $\kappa_{\text{root}} \to \infty$, it grows completely stationary. In this study, we use $c_{\text{root}} = 0.8382$ MPa and $\kappa_{\text{root}} = 8949$ MPa cm^{-2}, which keep the aortic root nearly still throughout the simulations. In future work, we plan to explore flexible models of the aortic root that allow for realistic distension.

G Stent Frame Structural Mechanics Model

The Evolut R's nitinol stent frame is modeled using a collection of Lagrangian points that are connected by a series of beams and springs,[6,15] as mentioned in the "Stent Frame" section of the main text. Each pair of adjacent points, say $\boldsymbol{X}_i$ and $\boldsymbol{X}_j$, along the stent's 30 vertical curves is connected by a linear spring that resists compression and extension. Defining $\boldsymbol{\chi}_i = \boldsymbol{\chi}(\boldsymbol{X}_i, t)$ and $\boldsymbol{\chi}_j = \boldsymbol{\chi}(\boldsymbol{X}_j, t)$, the forces generated on the Lagrangian points by a single spring are:

$$\begin{aligned} \boldsymbol{F}_{\text{spring}}(\boldsymbol{X}_i, t) &= k_{\text{s}} \left(1 - \tfrac{R_{\text{l}}}{\|\boldsymbol{\chi}_i - \boldsymbol{\chi}_j\|}\right) \cdot (\boldsymbol{\chi}_j - \boldsymbol{\chi}_i) \\ \boldsymbol{F}_{\text{spring}}(\boldsymbol{X}_j, t) &= k_{\text{s}} \left(1 - \tfrac{R_{\text{l}}}{\|\boldsymbol{\chi}_i - \boldsymbol{\chi}_j\|}\right) \cdot (\boldsymbol{\chi}_i - \boldsymbol{\chi}_j), \end{aligned} \tag{S6}$$

in which R_{l} and k_{s} are the resting length and spring constant, respectively. Additionally, each triplet of successive points, say $\boldsymbol{X}_i$, $\boldsymbol{X}_j$, and $\boldsymbol{X}_k$, along the stent's vertical curves is connected by a beam that resists bending. The bending-resistant forces generated on the Lagrangian points by a single beam are:

$$\begin{aligned} \boldsymbol{F}_{\text{beam}}(\boldsymbol{X}_i, t) &= -\,k_{\text{b}} \left(\boldsymbol{\chi}_k - 2\boldsymbol{\chi}_j + \boldsymbol{\chi}_i - \boldsymbol{C}\right) \\ \boldsymbol{F}_{\text{beam}}(\boldsymbol{X}_j, t) &= 2k_{\text{b}} \left(\boldsymbol{\chi}_k - 2\boldsymbol{\chi}_j + \boldsymbol{\chi}_i - \boldsymbol{C}\right) \\ \boldsymbol{F}_{\text{beam}}(\boldsymbol{X}_k, t) &= -\,k_{\text{b}} \left(\boldsymbol{\chi}_k - 2\boldsymbol{\chi}_j + \boldsymbol{\chi}_i - \boldsymbol{C}\right), \end{aligned} \tag{S7}$$

in which k_b is the beam constant, and $\boldsymbol{C}$ is the vector of preferred curvatures of the beam, given by

$$\boldsymbol{C} = \boldsymbol{X}_{k_\mathrm{B}} - 2\boldsymbol{X}_{j_\mathrm{B}} + \boldsymbol{X}_{i_\mathrm{B}}, \tag{S8}$$

with the subscript B signifying the base or preferred configuration of the three points. Each Lagrangian point is subject to the sum of forces generated from the collection of beams and springs that include it. In combination, the forces from this network of beams and springs hold the discrete stent frame together and penalize it towards its expanded equilibrated configuration. In this study, we use $k_\mathrm{s} = 1.0 \times 10^7$ dyne/cm and $k_\mathrm{b} = 1.0 \times 10^7$ dyne/cm, which allows us to crimp the stent but also causes it to self-expand upon release and maintain its deployed configuration inside the aortic root throughout the cardiac cycle under realistic driving and loading conditions. Resultant forces on each Lagrangian point are spread to the background grid using a four-point B-spline kernel function.[11] The same kernel function is used to interpolate the velocity from the background grid to the Lagrangian points.

H Parameter Fitting for the Boundary Condition Models

The parameters for our time-dependent elastance-based left heart model given in the "Fluid Model and Boundary Conditions" section of the main text are fit to experimental measurements of human LVOT pressures (P_LVOT) and aortic flow rates (Q_LVOT) from Murgo et al.[14] by taking the experimental measurements of Q_LVOT as input to our reduced-order model described by (7), (8), (9), and (10) in the main text and comparing the resulting values of P_LVOT to its experimental values. Then, the downstream Windkessel model parameters that coincide with this upstream model are also fit to experimental measurements of human aortic pressures (P_Ao) and aortic flow rates (Q_Ao) from Murgo et al. by taking the experimental measurements of Q_Ao as input to the Windkessel model, described by (5) and (6) in the main text, and comparing the resulting values of P_Ao to its experimental values. We calculate the best-fit parameters to data from Murgo et al. for a "Type A" beat for this upstream model and its corresponding downstream counterpart independently, using MATLAB's fmincon, a nonlinear optimization tool. Figure S2 compares the pressure (Fig. S2a) and flow rate (Fig. S2b) waveforms between experimental measurements from Murgo et al. and the healthy native valve model without the Evolut R device. The mean systolic transvalvular pressure difference in the simulated results is −1.77 mmHg, and the stroke volume is 92.92 mL, yielding a cardiac output of 6.55 L/min. This simulation of a healthy native valve establishes a baseline in order to create a stenotic version of the native aortic valve to replace with the Evolut R TAVR device.

I Immersed Finite Element-Difference Formulation

I.1 Continuum Equations

The IFED formulation relies on the combination of an Eulerian (spacial) coordinate system with Lagrangian (material) coordinate systems for the immersed structures. In

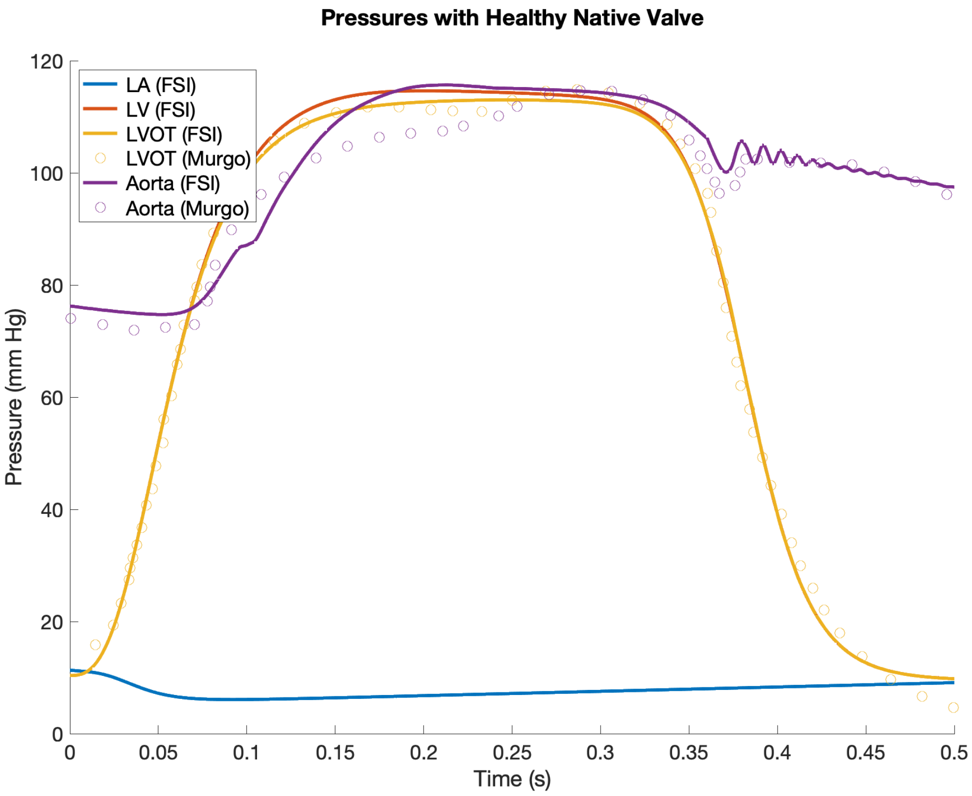

(a)

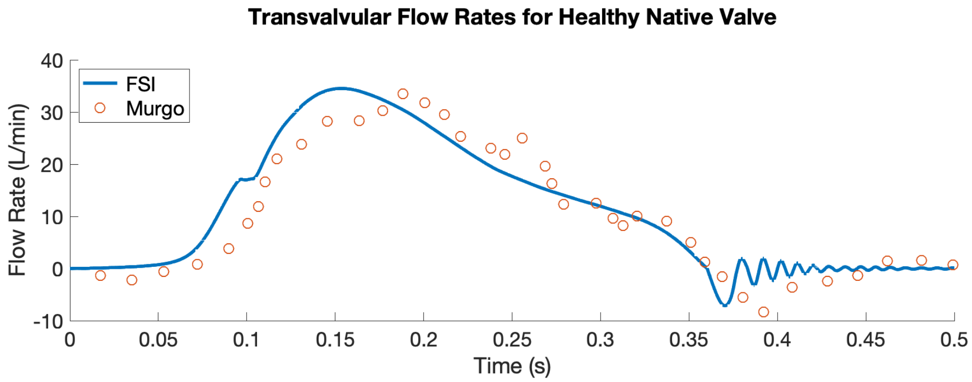

(b)

Figure S2: Comparisons of pressure and flow rate waveforms between experimental measurements from Murgo et al.[14] and the healthy native valve model using driving conditions based on the time-dependent elastance-based left heart model, as given by (7), (8), (9), and (10) in the main text. (a) Simulated left atrial (LA), left ventricular (LV), left ventricular outflow tract (LVOT), and aortic pressure waveforms compared to experimental measurements from Murgo et al. The mean systolic transvalvular pressure difference in the simulated results is -1.77 mmHg. (b) Simulated transvalvular flow rate compared to experimental measurements from Murgo et al. The stroke volume in the simulated results is 92.92 mL, yielding a cardiac output of 6.55 L/min.

particular, an Eulerian description is used for momentum conservation, viscosity, and incompressibility of the system, whereas a Lagrangian description is used to track the immersed structures' deformations, stresses, and resulting forces. Integral equations with delta function kernels couple the two descriptions together.

We describe the three-dimensional computational domain in terms of fixed Eulerian physical coordinates $\boldsymbol{x} = (x_1, x_2, x_3) \in \Omega \subset \mathbb{R}^3$. Further, we partition the domain into time-dependent fluid and solid regions $\Omega = \Omega_t^{\mathrm{f}} \cup \Omega_t^{\mathrm{s}}$. In contrast, we use Lagrangian reference coordinates at time $t = 0$, $\boldsymbol{X} = (X_1, X_2, X_3) \in \Omega_0^{\mathrm{s}}$, to describe the structures, with $\boldsymbol{N}(\boldsymbol{X}) \in \partial\Omega_0^{\mathrm{s}}$ the outward unit normal vector to the reference configuration of the solid region at material point $\boldsymbol{X}$. The time-dependent mapping $\boldsymbol{\chi} : (\Omega_0^{\mathrm{s}}, t) \mapsto \Omega_t^{\mathrm{s}} \subset \Omega$ relates the reference coordinates to current physical coordinates, determining the solid subdomain at time t. Thus, $\boldsymbol{\chi}(\boldsymbol{X}, t)$ gives the physical position of material point $\boldsymbol{X}$ (from the reference configuration) at time t. ρ and μ are the mass density and viscosity. $\boldsymbol{u}(\boldsymbol{x}, t)$ and $p(\boldsymbol{x}, t)$ are the Eulerian velocity and pressure fields, and $\boldsymbol{f}(\boldsymbol{x}, t)$ is the Eulerian elastic force density. $\mathbb{P}(\boldsymbol{X}, t)$ is the first Piola-Kirchhoff elastic stress tensor of the immersed structure, and $\delta(\boldsymbol{x}) = \prod_{i=1}^{3} \delta(x_i)$ is the three-dimensional Dirac delta function. The equations of motion for the fluid-structure system are[5,13,18,19,22,23]

$$\rho \frac{\mathrm{D}\boldsymbol{u}}{\mathrm{D}t}(\boldsymbol{x}, t) = -\nabla p(\boldsymbol{x}, t) + \mu \nabla^2 \boldsymbol{u}(\boldsymbol{x}, t) + \boldsymbol{f}(\boldsymbol{x}, t), \tag{S9}$$

$$\nabla \cdot \boldsymbol{u}(\boldsymbol{x}, t) = 0, \tag{S10}$$

$$\boldsymbol{f}(\boldsymbol{x}, t) = \int_{\Omega_0^s} \nabla_{\boldsymbol{X}} \cdot \mathbb{P}(\boldsymbol{X}, t)\, \delta(\boldsymbol{x} - \boldsymbol{\chi}(\boldsymbol{X}, t))\, \mathrm{d}\boldsymbol{X} - \int_{\partial\Omega_0^s} \mathbb{P}(\boldsymbol{X}, t)\, \boldsymbol{N}(\boldsymbol{X})\, \delta(\boldsymbol{x} - \boldsymbol{\chi}(\boldsymbol{X}, t))\, \mathrm{d}A, \tag{S11}$$

$$\frac{\partial \boldsymbol{\chi}}{\partial t}(\boldsymbol{X}, t) = \int_{\Omega} \boldsymbol{u}(\boldsymbol{x}, t)\, \delta(\boldsymbol{x} - \boldsymbol{\chi}(\boldsymbol{X}, t))\, \mathrm{d}\boldsymbol{x} = \boldsymbol{u}(\boldsymbol{\chi}(\boldsymbol{X}, t), t), \tag{S12}$$

in which $\mathrm{D}/\mathrm{D}t = \partial/\partial t + \boldsymbol{u}\cdot\nabla$ is the material derivative. This formulation automatically treats the immersed structure as exactly incompressible because $\partial\boldsymbol{\chi}/\partial t(\boldsymbol{X}, t) = \boldsymbol{u}(\boldsymbol{\chi}(\boldsymbol{X}, t), t)$ and $\nabla \cdot \boldsymbol{u}(\boldsymbol{x}, t) = 0$. Further, structures cannot interpenetrate because $\boldsymbol{u}(\boldsymbol{x}, t)$ is the common velocity field for both the fluid and solid regions and, due to viscosity, is continuous at the fluid-structure interfaces. The latter property is especially useful for our study because it provides an implicit contact model for the separate structures that interact in our model, including the native and Evolut R leaflets, the stent frame, and the aortic root.

I.2 Numerical Approximations

We discretize the computational domain Ω using an adaptively refined block-structured Cartesian grid. Although we employ local adaptive mesh refinement with multiple nested levels of Cartesian grid patches, we do not require body-conforming discretizations around the immersed bodies. Spacial resolution is increased dynamically near fluid-structure interfaces and in areas of detailed flow features, such as in areas of recirculation in the aortic sinuses and where vortices are shed from the tips of the valve leaflets. A staggered grid is used for the incompressible Navier-Stokes solver.[3] Additionally, we approximate the singular Dirac

delta function $\delta(\boldsymbol{x})$ with a regularized delta function $\delta_h(\boldsymbol{x})$. Similar to the continuous case, the three-dimensional regularized delta function is the tensor product of one-dimensional regularized delta functions, $\delta_h(\boldsymbol{x}) = \prod_{i=1}^{3} \delta_h(x_i)$. Here, the one-dimensional regularized delta function is constructed by a basic kernel function $\delta_h(x) = (\varphi\,(x/h))/h$.

In this study, we use different regularized delta functions for the rigid aortic root than for the flexible structures, such as the native valve leaflets and the porcine pericardial tissue that composes parts of the Evolut R. This decision follows the approach of Lee et al.[12] that is analyzed comprehensively by Lee and Griffith.[11] For the rigid aortic root, we use a piecewise-linear (PWL) kernel function given by

$$\varphi^{\mathrm{PWL}}(r) = \begin{cases} 1 - r, & 0 \le r < 1, \\ 0, & 1 \le r, \end{cases} \tag{S13}$$

and, for the more flexible valve leaflets, we use a three-point B-spline (BS3) kernel function given by

$$\varphi^{\mathrm{BS3}}(r) = \begin{cases} \frac{3}{4} - r^2, & 0 \le r < 0.5, \\ \frac{9}{8} - \frac{3}{2}r + \frac{1}{2}r^2, & 0.5 \le r \le 1.5, \\ 0, & 1.5 \le r. \end{cases} \tag{S14}$$

The use of regularized delta functions impacts the implicit contact model between structures described previously. For example, the "gap" between the valve leaflets when closed reflects the width of the regularized delta function across grid cells; however, despite the space between leaflets, the valve is effectively closed during diastolic loading and does not allow leaks. This characteristic of the IFED model eliminates the need for an explicit contact model to simulate contact between structures, such as the native and Evolut R leaflets, the stent frame, and the aortic root.

J Numerical Discretizations of the Model

Our IFED formulation approximates Eulerian variables using a block-structured Cartesian grid with adaptive mesh refinement, as described above in Supplemental Materials Section I.2, but we approximate Lagrangian variables using unstructured FE meshes that conform to the geometries of the immersed structures.[5] In this study, the computational domain is a cube, sized to embed the aortic root geometry described in the "Anatomical and Device Geometries" section of the main text, so it measures 7.68 cm on each side. We use a locally refined Cartesian grid with an effective fine-grid resolution of 0.4 mm that corresponds to a uniform $N \times N \times N$ grid with $N = 192$. The finite element mesh for the aortic root uses linear tetrahedral (four-node) elements, while the meshes for the native aortic valve leaflets, Evolut R pericardial leaflets, and Evolut R sealing skirt are composed of quadratic tetrahedral (10-node) elements. All meshes are generated using Coreform Cubit (Computational Simulation Software, LLC, American Fork, UT, USA), which is a software application based on the CUBIT software developed by Sandia National Laboratory. The maximum element edge length for each of the meshes is 0.4 mm, approximately coinciding with the fine-grid resolution of the background Cartesian grid. Since we use an explicit time

stepping algorithm, the largest time step size used is $\Delta t = 2.5\,\mu$s, which we systematically reduce to avoid temporal instabilities.

K Software Infrastructure

The FSI simulations in this study are performed using the IBAMR software infrastructure, which is a distributed-memory parallel processing implementation of the IB method with adaptive mesh refinement (AMR).[4,9] IBAMR uses SAMRAI[8] to manage Cartesian grid discretization, libMesh[10] to manage finite element discretization, and PETSc[2] for a linear solver infrastructure.

L Simulation Environment

We run our FSI simulations using 64 processor cores on the *Longleaf* cluster managed by University of North Carolina Information Technology Services Research Computing. Under these specifications, our FSI simulations take about five days to compute a full cardiac cycle without the Evolut R device and about fourteen days to compute a full cardiac cycle after deployment of the Evolut R. This study focuses on developing a model of the Evolut R rather than on parallel performance analysis; however, there is an ongoing effort to improve the parallelization of the IBAMR software library, and the effect of this work on future models will be analyzed in future studies.

Supplemental References

[1] Aazami, M. H. and M. Salehi. The Arantius nodule: a ‘stress-decreasing effect’. J. Heart Valve Dis. 14:565–566, 2005.

[2] Balay, S., S. Abhyankar, M. F. Adams, J. Brown, P. Brune, K. Buschelman, L. Dalcin, A. Dener, V. Eijkhout, W. D. Gropp, D. Karpeyev, D. Kaushik, M. G. Knepley, D. A. May, L. C. McInnes, R. T. Mills, T. Munson, K. Rupp, P. Sanan, B. F. Smith, S. Zampini, H. Zhang, and H. Zhang. PETSc users manual. Technical Report ANL-95/11 - Revision 3.15, Argonne National Laboratory, 2021.

[3] Griffith, B. E. An accurate and efficient method for the incompressible Navier–Stokes equations using the projection method as a preconditioner. J. Comput. Phys. 228:7565–7595, 2009.

[4] Griffith, B. E., R. D. Hornung, D. M. McQueen, and C. S. Peskin. An adaptive, formally second order accurate version of the immersed boundary method. J. Comput. Phys. 223:10–49, 2007.

[5] Griffith, B. E. and X. Luo. Hybrid finite difference/finite element immersed boundary method. Int. J. Numer. Method Biomed. Eng. 33:e2888, 2017.

[6] Griffith, B. E., X. Luo, D. M. McQueen, and C. S. Peskin. Simulating the fluid dynamics of natural and prosthetic heart valves using the immersed boundary method. Int. J. Appl. Mech. 1:137–177, 2009.

[7] Hasan, A., E. M. Kolahdouz, A. Enquobahrie, T. G. Caranasos, J. P. Vavalle, and B. E. Griffith. Image-based immersed boundary model of the aortic root. Med. Eng. Phys. 47:72–84, 2017.

[8] Hornung, R. D. and S. R. Kohn. Managing application complexity in the SAMRAI object-oriented framework. Concurr. Comput. 14:347–368, 2002.

[9] IBAMR. Immersed Boundary method Adaptive Mesh Refinement Software Infrastructure. `https://ibamr.github.io/`, accessed May 1, 2022.

[10] Kirk, B. S., J. Peterson, R. Stogner, and G. Carey. libMesh: A C++ library for parallel adaptive mesh refinement/coarsening simulations. Eng. Comput. 22:237–254, 2005.

[11] Lee, J. H. and B. E. Griffith. On the lagrangian-eulerian coupling in the immersed finite element/difference method. J. Comput. Phys. 457, 2022.

[12] Lee, J. H., A. D. Rygg, E. M. Kolahdouz, S. Rossi, S. M. Retta, N. Duraiswamy, L. N. Scotten, B. A. Craven, and B. E. Griffith. Fluid-structure interaction models of bioprosthetic heart valve dynamics in an experimental pulse duplicator. Ann. Biomed. Eng. 48:1475–1490, 2020.

[13] Liu, W. K., Y. Liu, D. Farrell, L. Zhang, X. S. Wang, Y. Fukui, N. Patankar, Y. Zhang, C. Bajaj, J. Lee, J. Hong, X. Chen, and H. Hsu. Immersed finite element method and its applications to biological systems. Comput. Methods Appl. Mech. Eng. 195:1722–1749, 2006.

[14] Murgo, J. P., N. Westerhof, J. P. Giolma, and S. A. Altobelli. Aortic input impedance in normal man: relationship to pressure wave forms. Circulation 62:105–116, 1980.

[15] Peskin, C. S. The immersed boundary method. Acta Numer. 11:479–517, 2002.

[16] Pham, T., F. Sulejmani, E. Shin, D. Wang, and W. Sun. Quantification and comparison of the mechanical properties of four human cardiac valves. Acta Biomater. 54:345–355, 2017.

[17] Sahasakul, Y., W. D. Edwards, J. M. Naessens, and A. Tajik. Age-related changes in aortic and mitral valve thickness: implications for two-dimensional echocardiography based on an autopsy study of 200 normal human hearts. Am. J. Cardiol. 62:424–430, 1988.

[18] Wang, X., C. Wang, and L. T. Zhang. Semi-implicit formulation of the immersed finite element method. Comput. Mech. 49:421–430, 2012.

[19] Wang, X. and L. T. Zhang. Interpolation functions in the immersed boundary and finite element methods. Comput. Mech. 45:321, 2009.

[20] Wong, J. and E. Kuhl. Generating fibre orientation maps in human heart models using Poisson interpolation. Comput. Methods Biomech. Biomed. Engin. 17:1217–1226, 2014.

[21] Yushkevich, P. A., J. Piven, H. C. Hazlett, R. G. Smith, S. Ho, J. C. Gee, and G. Gerig. User-guided 3D active contour segmentation of anatomical structures: significantly improved efficiency and reliability. Neuroimage 31:1116–1128, 2006.

[22] Zhang, L. and M. Gay. Immersed finite element method for fluid-structure interactions. J. Fluids Struct. 23:839–857, 2007.

[23] Zhang, L., A. Gerstenberger, X. Wang, and W. K. Liu. Immersed finite element method. Comput. Methods Appl. Mech. Eng. 193:2051–2067, 2004.
